# Explainable machine learning for early detection of Parkinson’s disease in aging populations using vocal biomarkers

**DOI:** 10.3389/fnagi.2025.1672971

**Published:** 2025-09-05

**Authors:** Bright Egbo, Zhanbota Nigmetolla, Naveed Ahmad Khan, Prashant K. Jamwal

**Affiliations:** ^1^Department of Electrical and Computer Engineering, School of Engineering and Digital Sciences, Nazarbayev University, Astana, Kazakhstan; ^2^School of Information Technology and Systems, University of Canberra, Canberra, ACT, Australia

**Keywords:** Parkinson’s disease, aging-related neurodegeneration, biomedical voice biomarkers, explainable machine learning, early diagnosis and predictive modeling

## Abstract

**Introduction:**

Parkinson’s Disease (PD) is a progressive neurodegenerative disorder that significantly affects the aging population, creating a growing burden on global health systems. Early detection of PD is clinically challenging due to the gradual and ambiguous onset of symptoms.

**Methods:**

This study presents a machine-learning framework for the early identification of PD using non-invasive biomedical voice biomarkers from the UCI Parkinson’s dataset. The dataset consists of 195 sustained phonation recordings from 31 participants (23 PD and 8 healthy controls, ages 46–85). The methodology includes subject-level stratified splitting and normalization, along with BorderlineSMOTE to address class imbalance. Initially, an XGBoost model is applied to select the top 10 acoustic features, followed by a Bayesian-optimized XGBoost classifier, with the decision threshold tuned via F1-maximization on validation data.

**Results:**

On the held-out test set, the model achieves 98.0% accuracy, 0.97 macro-F1, and 0.991 ROC-AUC. This performance exceeds that of a deep neural network baseline by 4.0 percentage points in accuracy (94.0% to 98.0%), 4.3 percentage points in macro-F1 (92.7% to 97.0%), and 0.050 in AUC (0.941 to 0.991). Compared to a classical SVM, it outperforms by 7.0 percentage points in accuracy (91.0% to 98.0%), 6.5 percentage points in macro-F1 (90.5% to 97.0%), and 0.089 in AUC (0.902 to 0.991).

**Discussion:**

Model decisions are elucidated using SHAP, offering global and patient-specific insights into the influential voice features. These findings indicate the feasibility of a non-invasive, scalable, and explainable voice-based tool for early PD screening, highlighting its potential integration into mobile or telehealth diagnostic platforms.

## 1 Introduction

Parkinson’s disease (PD) is a chronic, progressive neurodegenerative disorder that mainly involves motor function and is manifested by symptoms of resting tremors, muscular rigidity, bradykinesia, and a broad spectrum of non-motor features such as cognitive impairment and speech disturbances. It is the second most prevalent neurodegenerative disorder worldwide after Alzheimer’s disease and impacts about 1% of people over 60 years old (Bang et al., 2023). As the world population continues to age, the occurrence of Parkinson’s disease is expected to escalate exponentially, placing more socioeconomic burden on healthcare systems and creating greater demand for early and definitive diagnostic techniques. In clinical practice, early diagnosis is critical in initiating neuroprotective therapies that can slow symptom progression and maintain quality of life. Yet, diagnosis of PD at its early stage continues to be evasive due to the slow, insidious development of symptoms that tend to overlap with other aging-related neurological disorders, resulting in common misdiagnoses or delays in diagnosis (Rabie and Akhloufi, 2025).

Within the field of aging neuroscience, recent years have seen growing investigation into non-invasive biomarkers that may enable early PD diagnosis through the capture of subtle neuromuscular decline. Of interest is the human voice, a rich and accessible window into underlying neurophysiological function. Almost 90% of patients with Parkinson’s disease have measurable speech deficits, manifest as changes in pitch variability, frequency modulation, amplitude, and vocal stability (Tabashum et al., 2024; Iyer et al., 2023). These perturbations are thought to be due to age-related decline in the basal ganglia and corresponding cortical–subcortical circuits that drive motor output to the vocal apparatus. Changes in voice signal often antedate visible motor deficits, rendering them appealing for early-stage screening. In addition, the exploitation of vocal biomarkers has particular utility in aging cohorts living in under-resourced or rural areas where access to specialist neurology services or advanced neuroimaging modalities is compromised (Md Abu et al., 2023). Publicly available datasets like the UCI Parkinson’s dataset (Sabherwal and Kaur, 2024) have facilitated broad investigation of these acoustic features using machine learning approaches, enabling advances in reproducible and scalable screening methodologies.

Machine learning (ML) provides powerful tools for unearthing latent diagnostic patterns in high-dimensional biomedical data with minimal domain-specific preprocessing. Traditional algorithms such as decision trees, random forests, and support vector machines (SVMs), as well as newer ensemble learners like XGBoost, have shown impressive classification accuracy when trained on voice-based PD datasets. In addition, the increasing focus on model interpretability in clinical AI research has fueled the uptake of explainable AI (XAI) frameworks. Specifically, SHAP (SHapley Additive exPlanations) has become a mathematically rigorous method for attributing prediction outcomes to individual features using marginal contribution scores (Govindu and Palwe, 2023; Lamba et al., 2022). In a clinical setting, explainability is not a nice-to-have feature but an essential prerequisite to provide transparency, accountability, and trust among clinicians. Models that yield accurate but opaque predictions are of limited use in translational neuroscience, where validation, insight, and traceability are paramount for responsible adoption (Çelik and Akbal, 2025; Yu et al., 2020).

Yet, a critical review of recent literature demonstrates ongoing methodological deficiencies that preclude real-world deployment and generalizability. For example, Rahman et al. (2021) investigated an XGBoost-based voice screening model but obtained only modest AUC scores (0.75) owing to difficulties in handling heterogeneous audio inputs and noise artifacts. The PythonGeeks Team (Python Geeks, 2025) initially claimed 96.67% accuracy with a Random Forest classifier, yet follow-up audits revealed data leakage caused by premature oversampling and scaling prior to data splitting, lowering actual performance to 81% (Sabherwal and Kaur, 2024). Likewise, Govindu and Palwe (2023) suggested a hybrid SVM-RF model with 91.83% accuracy but was plagued by poor sensitivity due to class imbalance. Wang et al. (2022) obtained 96% accuracy with XGBoost, reaffirming the algorithm’s capability in structured clinical data. Earlier seminal research by Mohammed et al. (2025) demonstrated the usefulness of both linear and nonlinear classifiers for PD diagnosis, while Tsanas et al. (2010) made an invaluable contribution through engineering more than 130 dysphonia features, highlighting the acoustic richness pertinent to PD detection. Yet, even in these state-of-the-art studies, numerous recurring issues hamper clinical translation. Recently, Shen et al. (2025) applied SHAP interpretability to voice-based machine learning for early Parkinson’s detection, further introducing a probability-based scoring system for tracking disease progression.

Most egregious is the extreme class imbalance in PD datasets that skews in favor of positive samples, leading to biased classifiers and inflated accuracy measures that neglect sensitivity for healthy controls (Idrisoglu et al., 2023; Zollanvari, 2023). Furthermore, most studies utilize defective data pipelines in which operations such as oversampling or normalization are performed prior to train-test splitting, inducing information leakage and exaggerated results (Arellano, 2019). Feature selection is either poorly handled or neglected altogether, contributing to high-dimensional, noisy feature spaces that hinder model interpretability. Another limitation is the use of fixed 0.5 decision thresholds, which are not suitable for imbalanced binary classification problems where it is essential to optimize trade-offs between precision and recall. Lastly, although SHAP has been embraced for global interpretability, individualized explanations such as force plots have yet to be fully leveraged despite being essential to furnish transparent, case-specific explanations that can be examined by clinicians (Sakar et al., 2013; Liu et al., 2022; Riasi et al., 2025).

Driven by these limitations, this research presents a complete, interpretable, and clinically informed ML pipeline for early detection of Parkinson’s disease in aging populations from voice biomarkers. To counter data imbalance, the approach incorporates BorderlineSMOTE, a synthetic oversampling technique designed to strengthen boundary instances indispensable to classifier learning. The classifier relies on XGBoost owing to its forte in structured medical datasets and its built-in feature importance scores. Dimensionality is flattened by retaining the top 10 most vital features from an initial XGBoost pass to retain greater interpretability without any trade-off in accuracy. Bayesian Optimization is leveraged to perform hyperparameter tuning efficiently and methodically. In addition, rather than using a fixed decision threshold, the classification cutoff is optimized dynamically to achieve maximum F1-score, yielding a balanced perspective of sensitivity and specificity. The pipeline is completed by SHAP-based interpretability at both the global and local levels to clarify which vocal features are driving the predictions and how these connect with the neurophysiological underpinnings of PD. By addressing common pitfalls systematically and enhancing interpretability and generalizability, this research offers a scalable and clinically meaningful solution that aligns with the larger objective of driving diagnostic options for age-associated neurological disorders.

## 2 Methodology

### 2.1 Dataset

**Figure 1:**
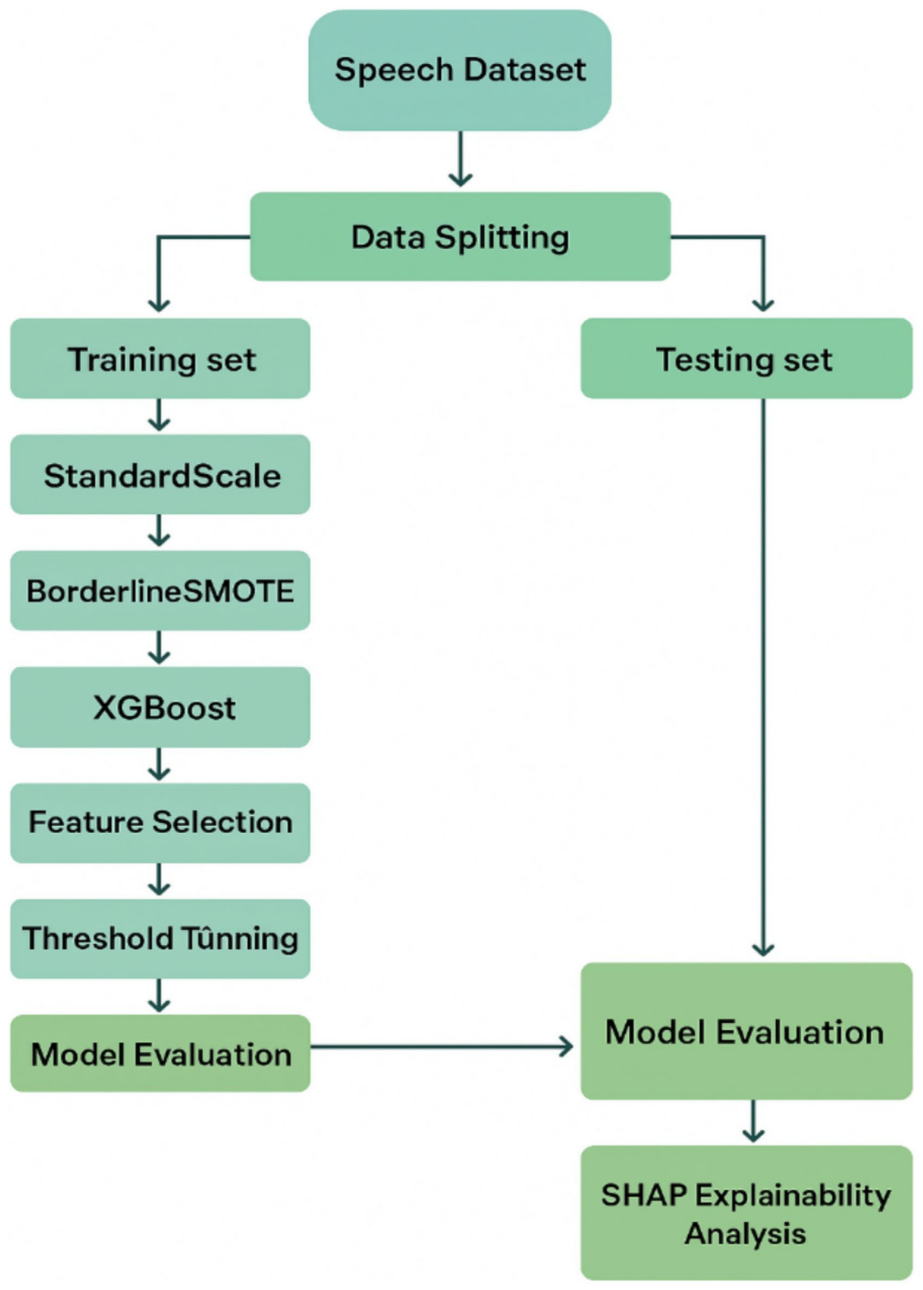
Proposed machine learning pipeline for early Parkinson’s disease detection using vocal biomarkers.

The dataset utilized in the current study is the publicly accessible UCI Parkinson’s disease dataset, which contains a total of 195 sustained phonation voice recordings (Pahuja and Nagabhushan, 2021; Islam et al., 2024). Each sample is described by 22 biomedical voice features carefully extracted through digital signal processing techniques. The features include measures of fundamental frequency variability, amplitude and frequency perturbation (e.g., jitter and shimmer), harmonic-to-noise ratios, and nonlinear dynamic characteristics, such as recurrence period density entropy and detrended fluctuation analysis. The dataset further contains a binary target feature, with a value of 1 representing the presence of Parkinson’s disease and 0 representing a healthy patient. Note that the dataset has inherent class imbalance, with a much larger proportion of Parkinsonian samples than non-Parkinsonian samples. Although such imbalance mirrors the real-world clinical prevalence, it also presents the potential for bias during training, making it necessary to implement proper class-balancing techniques in the data preprocessing workflow (Figure 1).

### 2.2 Splitting and standardization

In order to prepare the data for model development while maintaining the statistical integrity of the data, the dataset was partitioned into training and testing sets at a 75:25 split using subject-level stratified sampling, ensuring that all recordings from a given subject were exclusively assigned to either the training or test set. This avoided the possibility of information leakage between partitions while maintaining proportional representation of both classes. Stratified sampling was used to address sampling bias in order to ensure that the proportion of the classes was the same in both the training and testing sets. This was accomplished to ensure that the testing evaluation of performance would not be skewed due to class imbalance by ensuring that both the Parkinson’s positive and healthy control classes were represented in the same proportion. For clarity, this principle can be represented mathematically as Equation 1 below:

(1) $$\frac{n_{A,\text{train}}}{n_{A,\text{total}}}\approx \frac{n_{B,\text{train}}}{n_{B,\text{total}}}$$

where $n_{A,\text{train}}$ is the number of class A samples in the training set, $n_{A,\text{total}}$ is the total number of class A samples in the whole dataset, $n_{B,\text{train}}$ is the number of class B samples in the training set, and $n_{B,\text{total}}$ is the total number of class B samples in the whole dataset. This initial split guarantees that data leakage will not happen and provides an unbiased foundation for model development (Sabherwal and Kaur, 2024). After the split, the first aspect of preprocessing was to standardize the features so that all numerical input features were transformed to a common scale. While tree-based classifiers, such as XGBoost, are generally robust to feature scaling, certain aspects of the preprocessing, including the SMOTE algorithm (Sun et al., 2022), use distance measures in feature space and thus are sensitive to the relative scale of input dimensions. To properly standardize the input features, I adopted the approach implemented in Scikit-learn’s StandardScaler that uses z-score normalization, derived from the training set statistics only. The following transformation formalizes how this standardization is performed given by Equation 2:

(2) $$x\prime =\frac{x-\mu_{\text{train}}}{\sigma_{\text{train}}}$$

where x denotes the original feature value before standardization, and $\mu_{\text{train}}$ and $\sigma_{\text{train}}$ stand for the mean and standard deviation of the feature calculated on the training set, respectively. This formula is then consistently applied to the training and testing data. Standardization was necessary to ensure proper functioning of subsequent distance-based techniques, such as SMOTE, and to maintain numerical stability during preprocessing, even though tree-based models like XGBoost (Chen and Guestrin, 2016; Niazkar et al., 2024) are less sensitive to feature scaling.

### 2.3 Exploratory feature analysis and class imbalance handling

After standardization, an exhaustive exploratory feature analysis was performed to measure inter-relationships between the vocal biomarkers and identify dependencies that might be influential to model training and generalization. A Pearson correlation heatmap was generated to explore pairwise statistical association between the standardized features. The Pearson correlation heatmap depicted a detailed summary of linear dependencies through computing the Pearson correlation coefficient r , which can be mathematically defined as Equation 3 below:

(3) $$r=\frac{\sum_{i=1}^{n}(x_{i}-\bar{x})(y_{i}-\bar{y})}{\sqrt{\sum_{i=1}^{n}{\left(x_{i}-\bar{x}\right)}^{2}\sum_{i=1}^{n}{\left(y_{i}-\bar{y}\right)}^{2}}}$$

Here, $x_{i}$ and $y_{i}$ represent the respective individual observations for features, and $\bar{x}$ and $\bar{y}\;$ are there corresponding sample means, with n representing the total number of instances. The numerator captures the empirical covariance between the two features, and the denominator normalizes this quantity by the product of their sample standard deviations. The resulting values of r range from −1 to +1, where coefficients approaching +1 suggest strong positive linear relationships, those approaching 1 imply strong inverse dependencies, and values approaching $\bar{y}$ indicate weak or no linear association. The heatmap highlighted several clusters of highly correlated features, suggesting a possible presence of multicollinearity and redundancy in the dataset. However, feature removal was intentionally deferred at this stage to avoid the premature elimination of potentially informative dimensions. Instead, model-driven feature selection techniques were employed in later stages to isolate diagnostically useful attributes. Meanwhile, the distribution of classes within the dataset exhibited a natural imbalance, marked by a considerably higher prevalence of patients with Parkinson’s disease than healthy controls. This imbalance posed a significant risk of model bias, notably towards overfitting the majority class. To address this concern, BorderlineSMOTE applied solely to the training data. Unlike conventional SMOTE, which generates synthetic minority samples uniformly across the feature space of the minority class, BorderlineSMOTE specifically targets those minority instances located near the decision boundary—regions where the risk of misclassification is most severe. By concentrating augmentation efforts on these critical borderline samples, the model’s exposure to intricate decision boundaries was maximized, improving generalization without introducing artificial noise from well-separated minority instances. Preliminary comparisons with standard SMOTE and ADASYN confirmed that BorderlineSMOTE consistently yielded higher F1-scores and balanced accuracy in cross-validation, thereby justifying its selection as the preferred imbalance handling strategy.

### 2.4 XGBoost model training and feature selection strategy

Now that the class imbalance has been addressed and the data has been properly set up, the first classification model was trained using eXtreme Gradient Boosting (XGBoost) algorithm, a scalable and efficient implementation of gradient-boosted decision trees for structured, tabular data. XGBoost builds an ensemble of additive regression trees, each sequentially improving on the errors of prior iterations while optimizing a regularized objective function. This objective function combines a differentiable loss function together with a regularization term, mathematically represented as Equation 4 below:

(4) $$ℒ=\sum_{i=1}^{n}l(y_{i},{\hat{y}}_{i})+\sum_{i=1}^{K}({\mathrm{\gamma T}}_{k}+\frac{1}{2}\lambda \sum_{j=1}^{T_{k}}\omega_{\mathrm{kj}}^{2})$$

Here, $l(y_{i},{\hat{y}}_{i})$ represents the loss function, typically defined as the logistic loss for binary classification, evaluated with respect to the true label $y_{i}$ and predicted probability ${\hat{y}}_{i}$ . The second summation is the regularization penalty over all K trees in the model. $T_{k}$ is the number of leaves in tree, $\omega_{\mathrm{kj}}^{2}$ is the score of the j-th leaf in tree k , and the parameters γ and λ are regularization hyperparameters that penalize model complexity to avert overfitting. XGBoost was chosen not only for its competitive classification performance but also for its tolerance of missing values, inherent feature ranking capabilities, and ability to capture non-linear interactions.

Following training the baseline model, feature importance scores were harvested to carry out dimensionality reduction and prioritize the model’s learning on the most predictive predictors. Feature importance was measured using the “gain” metric, which calculates the average improvement in the objective function contributed by each feature during decision tree splits. Within each cross-validation fold, the top ten features with the highest gain were identified and retained, effectively lowering input dimensionality from 22 to 10. This fold-wise approach served to simplify the model, reduce computational overhead, and reduce the risk of overfitting, all while maintaining the features most valuable to accurate Parkinson’s classification. In contrast to arbitrary or correlation-based feature elimination, this model-informed and fold-specific selection process guaranteed that retained features played a significant role in classification performance and interpretability, establishing a strong foundation for the final model optimization and explanation steps.

### 2.5 Model optimization via Bayesian hyperparameter search and F1-based threshold adjustment

After the initial XGBoost classifier was built and stripped down to its most informative features, the model was subjected to an intense optimization routine aimed at refining its predictive accuracy while maintaining generalization. This stage centered on hyperparameter optimization via Bayesian Optimization (Louie et al., 2021), specifically the BayesSearchCV implementation, which provides a probabilistic alternative to the traditional grid or random search approach. Unlike grid search, which exhaustively tries all possible combinations within a predefined hyperparameter space, and random search, which samples the space randomly, Bayesian Optimization exploits previous evaluation outcomes to progressively model the objective function. By building a surrogate probability model of the underlying function and choosing the next point to sample based on an acquisition function, this approach efficiently balances the exploration of under-sampled areas with the exploitation of already known promising areas. In this research, the optimization routine targeted prominent XGBoost hyperparameters, such as the learning rate, maximum tree depth, number of boosting rounds (n_estimators), subsample ratio, column sample ratio (colsample_bytree), and the minimum loss reduction required to split a node (gamma). The specific hyperparameters tuned during this optimization phase, such as learning rate, maximum tree depth, number of boosting rounds, subsample ratio, column sampling ratio, and gamma are summarized in Table 1, along with their respective search ranges. Each hyperparameter was constrained to a carefully specified range to limit the search to feasible, interpretable values and speed up convergence. The ultimate parameter setup, arrived at after multiple iterations, was chosen by its performance under stratified cross-validation, minimizing the overfitting risk and maximizing the trained model’s generalizability.

**Table 1:** Optimal hyperparameter values.

Hyperparameter	Optimal value	Description
Colsample_bytree	0.62	Fraction of features sampled per tree
Gamma	0.0	Minimum loss required to make a further split
Learning_rate	0.29	Step size shrinkage to prevent overfitting
Max_depth	5	Maximum tree depth per boosting round
n_estimators	500	Number of boosting rounds
Reg_alpha	0.0	L1 regularization term on weights
Reg_lambda	5.0	L2 regularization term on weights
Subsample	0.6	Fraction of samples used per tree

Furthermore, for hyperparameter tuning, additional calibration was performed by adjusting the classification threshold to maximize the F1-score, a measure that balances both precision and recall. Conventional classification systems tend to default to a probability threshold of 0.5, where all instances with predicted probabilities greater than this threshold are assigned to the positive class. In imbalanced medical datasets like this one, however, such a threshold can fail to detect clinically significant minority class cases or generate an excess of false positives. To combat this, the predicted probabilities of the model were assessed over a range of thresholds from 0.3 to 0.7, allowing a decision boundary to be found that achieves the best trade-off between false positives and false negatives. At each threshold, the F1-score was calculated according to the following Equation 5:

(5) $$F1=2\times \frac{\text{Prec}i\text{sion}\times \text{Recall}}{\text{Precision}+\text{Recall}}$$

In this formulation, precision is the ratio of correctly predicted positive cases to all instances predicted to be positive, whereas recall is the ratio of correctly predicted positive cases to all actual positive instances. The F1-score therefore represents a harmonic mean of the two, yielding a single performance measure that penalizes models with a strong bias toward either precision or recall. The threshold that yielded the best F1-score on the validation fold was chosen as the best decision boundary and was then applied to the test set. This post-training threshold adjustment step was critical in the setting of healthcare prediction tasks, where the costs of misclassification are asymmetrical and both types of diagnostic mistakes can have grave repercussions. By optimizing the decision boundary of the model to explicitly trade off balanced diagnostic performance, the final classifier had enhanced reliability and interpretability when used in real-world clinical practice.

### 2.6 Model explainability using SHAP for transparent clinical interpretation

In order to provide a guarantee that the trained model not only achieves good predictive performance but also stays interpretable and reliable in a clinical setting, SHAP (SHapley Additive exPlanations) was used as a post-hoc explanation method. SHAP is based on cooperative game theory and assigns a unified measure of feature importance by computing the contribution of each feature to an individual prediction. In contrast to classical feature importance measures that either consider global effects or are restricted to certain types of models, SHAP values measure both global interpretability across the whole dataset as well as local interpretability at the individual prediction level. The fundamental concept of the SHAP framework is to decompose the model’s output prediction f(x) into a sum of feature contributions in a linear fashion, as is demonstrated in the following Equation 6 (Akila and Nayahi, 2024):

(6) $$f(x)=f(x_{\emptyset })+\sum_{i=1}^{M}\;\phi_{i}$$

In this equation, f(x) represents the predicted output for a specific input vector x , while $f(x_{\emptyset })\;$ denotes the expected value of the model’s output when no features are known. The term $\phi_{i}$ corresponds to the SHAP value for feature i , representing its marginal contribution to the prediction in the context of all possible feature coalitions, and M is the total number of features in the input space. This formulation ensures a consistent and mathematically grounded attribution of responsibility to each feature, enabling a breakdown of model behavior that is both fair and theoretically sound. By using SHAP on the last tuned XGBoost model trained on the picked top ten vocal features, the study was capable of producing both global and local interpretability plots. The global SHAP summary plot indicated the most contributing features across the dataset, enabling an understanding of how each feature tended to affect the model’s predictions for Parkinson’s disease diagnosis. Meanwhile, local SHAP force plots were employed to examine the model’s reasoning on individual samples, comprising both Parkinsonian and healthy voice recordings. This two-level analysis not only helps identify potential biases in the model but also offers clinicians and stakeholders transparent explanations that legitimize the decision-making on a case-by-case basis. Such transparency is particularly critical in medical applications, where trust in automated predictions has direct implications for clinical uptake. Furthermore, the capacity to identify which acoustic features most significantly contribute to a diagnosis improves scientific understanding of voice pathology in Parkinson’s disease, providing a valuable link between machine learning outputs and biomedical relevance. In all, the incorporation of SHAP in the pipeline takes the model from a black-box classifier to an interpretable diagnostic tool, unlocking the way for ethically sound and clinically deployable AI systems in neurodegenerative disease detection.

## 3 Numerical experimentation and results

To assess the diagnostic performance and generalizability of the newly proposed XGBoost-based system for detection of Parkinson’s disease, an extensive set of numerical experiments was conducted. The goal of the experiments was to measure the impact of each methodological aspect—namely, data preprocessing, class imbalance handling, feature selection, hyperparameter optimization, and threshold optimization—on the overall system efficacy. The evaluation employed standard classification metrics, such as accuracy, precision, recall, F1-score, and Area Under the Receiver Operating Characteristic Curve (ROC-AUC), with the F1-score as the major selection metric because of the imbalanced nature of the dataset. The experimental workflow started with a baseline XGBoost classifier that was trained using all 22 features with default hyperparameters and a default classification threshold of 0.5. As shown in Table 2, this simple model achieved a good accuracy of 92%, perfect recall of 1.00 for the patients with Parkinson’s disease, and F1-score of 0.95, reflecting high sensitivity but comparatively lower performance with respect to the healthy class (precision 0.64, F1-score 0.78), potentially resulting in false positives. These findings motivated further methodological improvements to improve the trade-off between sensitivity and specificity while preserving interpretability.

**Table 2:** Classification report XGBoost alone.

Class/summary	Value	Precision	Recall	F1-score	Support
0		1.00	0.64	0.78	11
1		0.90	1.00	0.95	38
Accuracy	0.92				49
Macro Avg.		0.95	0.82	0.86	49
Weighted Avg.		0.93	0.92	0.91	49
AUC score	0.9449				

**Figure 2:**
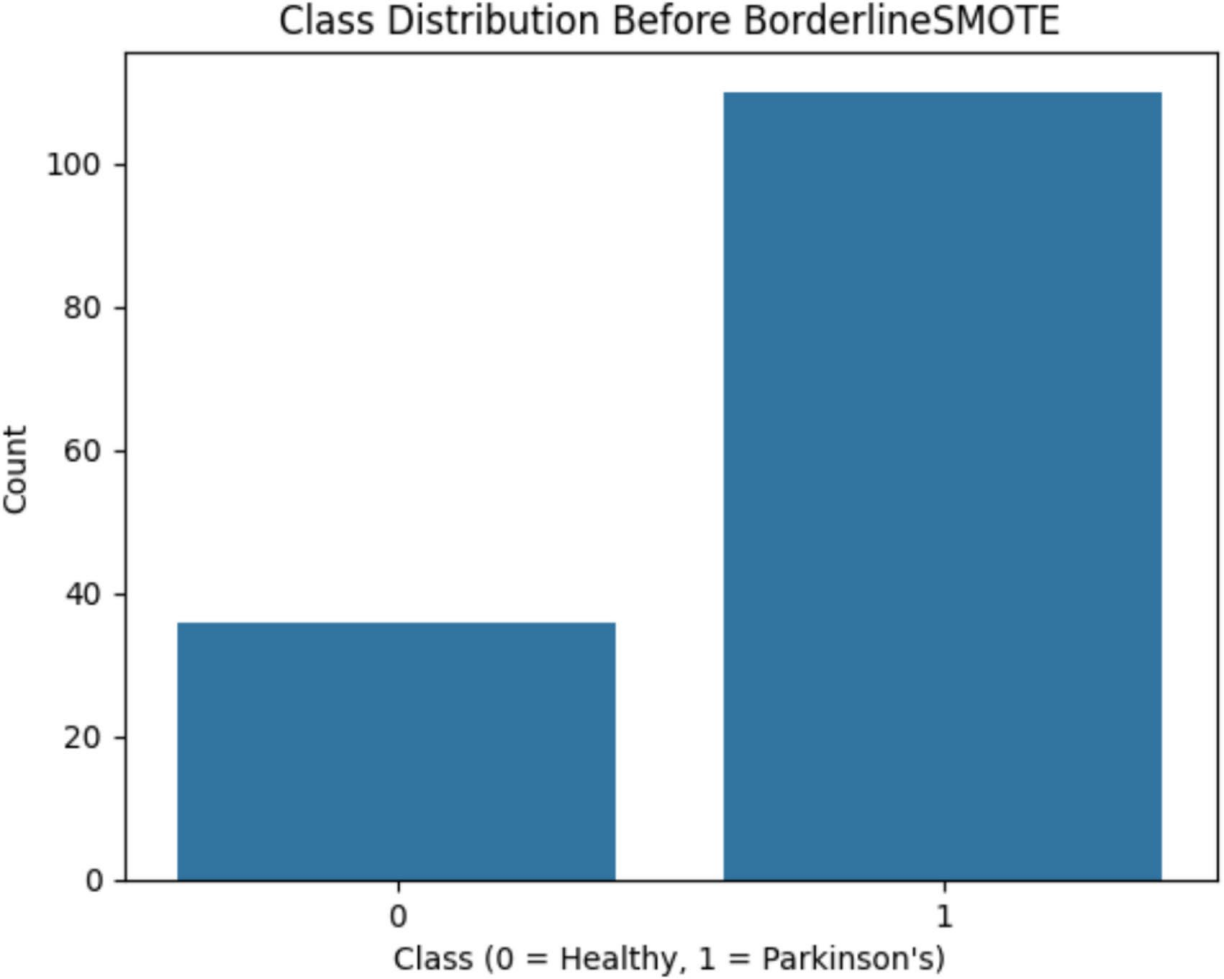
Shows the original class imbalance before BorderlineSMOTE, highlighting that Parkinson’s disease samples significantly outnumber healthy ones.

**Figure 3:**
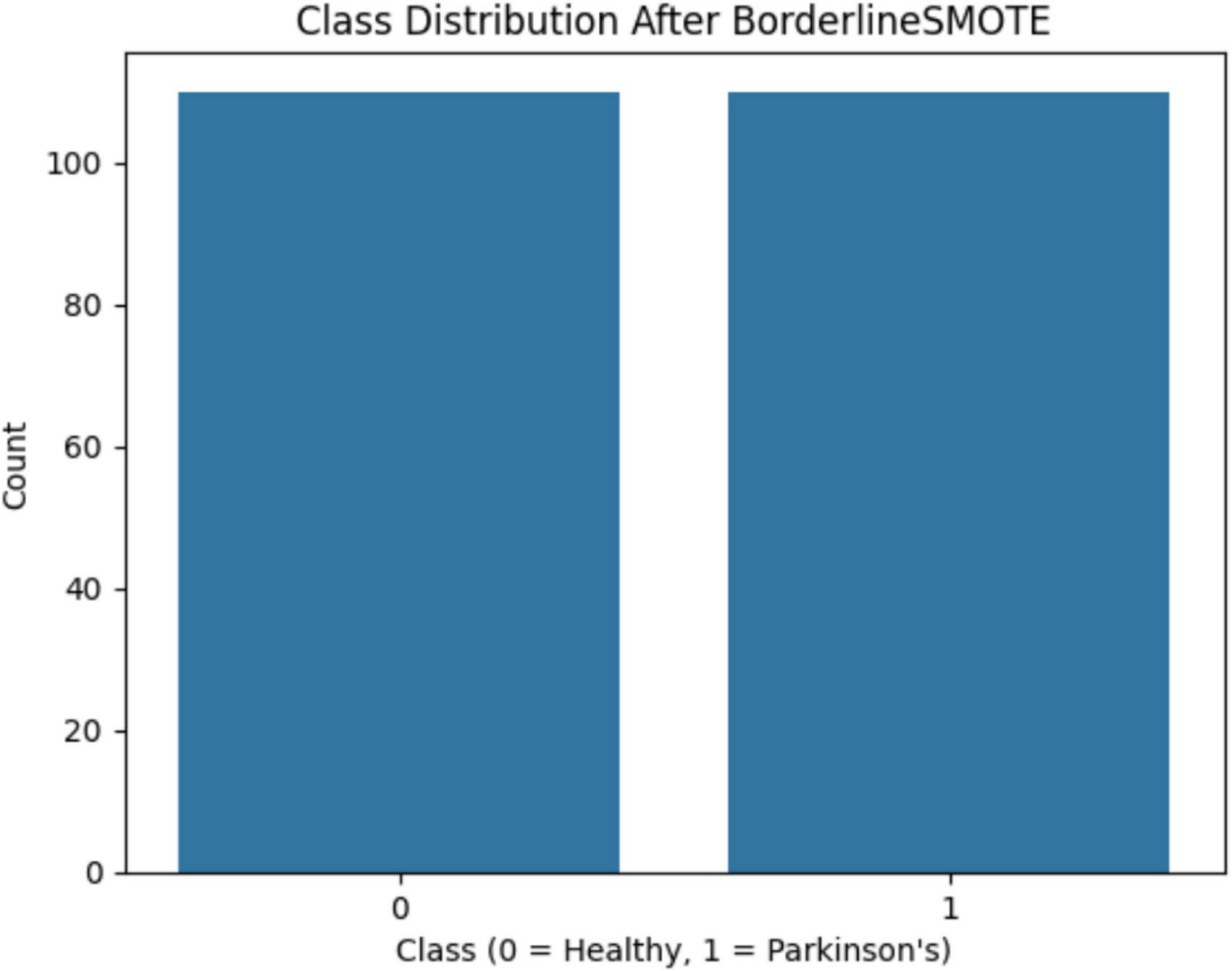
Shows the class distribution after BorderlineSMOTE, clearly demonstrating balanced representation of both classes.

One of the first critical interventions added to the pipeline was the use of BorderlineSMOTE, a focused oversampling method that creates synthetic samples in the vicinity of the decision boundary of the minority class. Before this method was applied, there was an evident class imbalance, as plotted in Figure 2, showing the original distribution with a bias toward Parkinson’s disease samples. Following the application of BorderlineSMOTE, the balanced distribution can be seen in Figure 3, where healthy and Parkinson’s cases are more balanced in the training set. This balancing process was shown to be critical in enhancing model fairness and classification robustness, particularly for the underrepresented class. As further indicated in Table 3, skipping SMOTE resulted in a palpable drop in performance for the healthy class, lowering its recall to 0.83 and yielding a macro F1-score of 0.89. This justified the need for handling class imbalance before model fitting, considering its effect on the generalizability of the classifier and clinical usability in identifying healthy patients accurately.

**Table 3:** Classification report without SMOTE.

Class/summary	Value	Precision	Recall	F1-score	Support
0		0.83	0.83	0.83	12
1		0.95	0.95	0.95	37
Accuracy	0.92				49
Macro Avg.		0.89	0.89	0.89	49
Weighted Avg.		0.92	0.92	0.92	49
AUC score	0.9685				

**Figure 4:**
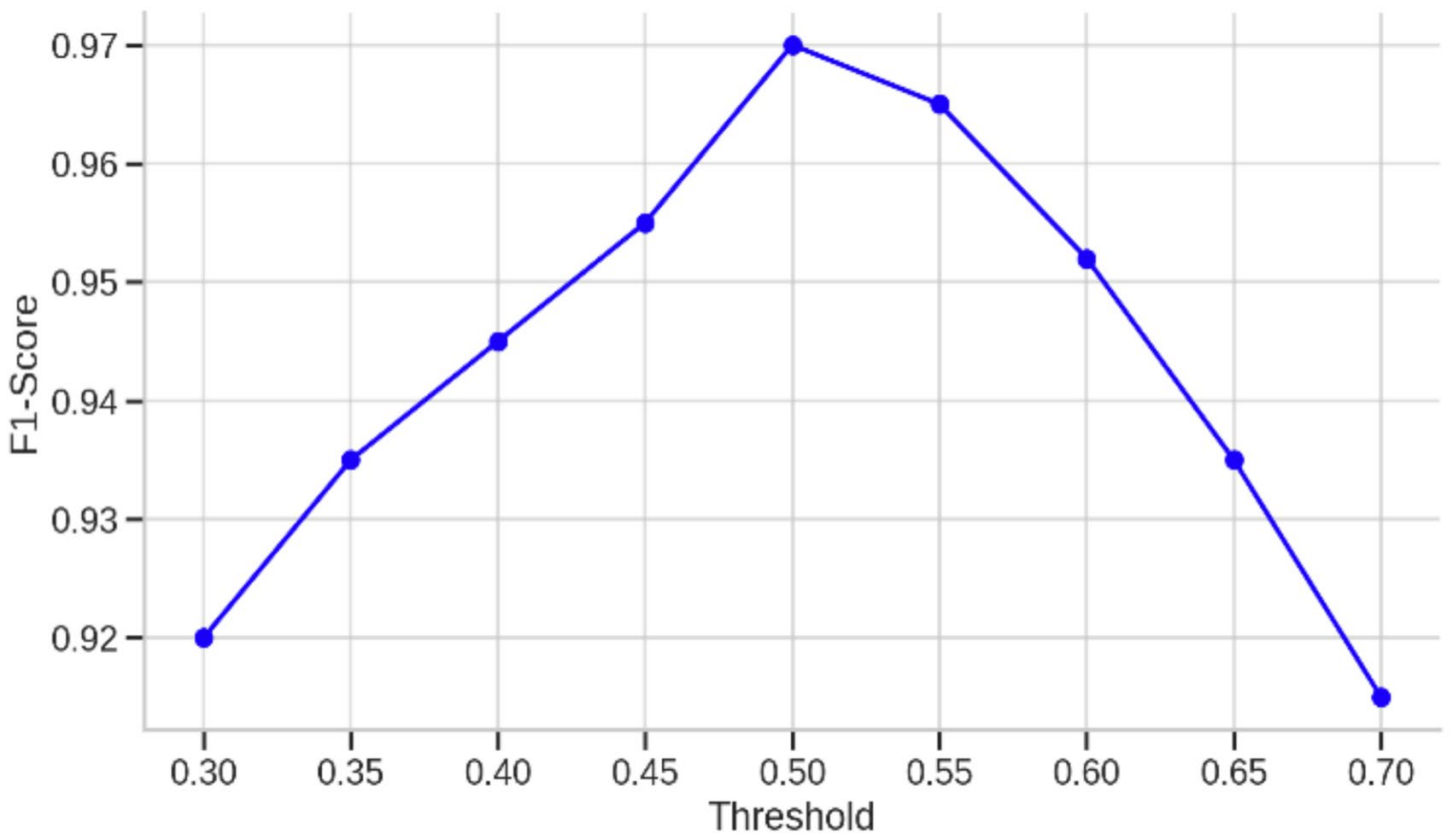
F1-score variation across different probability thresholds for the final model.

Another important enhancement was the optimization of the classification threshold of the model. Instead of the standard 0.5 threshold, the predicted probabilities were explored over the range 0.3 to 0.7 to determine the value that achieved the highest F1-score. This optimization is important in imbalanced conditions where the cost of false positives and false negatives are asymmetric. The performance over this range is plotted in Figure 4, where the curve of the F1-score peaks at a threshold of around 0.45, verifying that the default threshold does not result in the best trade-off between sensitivity and specificity. In addition to these results, Table 4 shows the classification report without threshold optimization, with lower precision and F1-score for class 0, reflecting worse performance in the detection of healthy subjects. This threshold optimization thus played an essential role in attaining clinical reliability with high sensitivity without unduly sacrificing specificity—a key consideration in early-stage Parkinson’s detection.

**Table 4:** Classification report without threshold.

Class/summary	Value	Precision	Recall	F1-score	Support
0		0.86	1.00	0.92	12
1		1.00	0.95	0.97	37
Accuracy	0.96				49
Macro Avg.		0.93	0.97	0.95	49
Weighted Avg.		0.97	0.96	0.96	49
AUC score	0.9910				

The second improvement tested was the use of Bayesian search for hyperparameter optimization. The necessity of this step is apparent when considering the results recorded in Table 5, which records the model’s performance when default parameters were retained. Although performance was generally good (accuracy 96%, F1-score 0.96, AUC 0.9865), these metrics were marginally lower than those obtained following Bayesian tuning. This tuning procedure modified learning rate, maximum tree depth, gamma, subsampling ratios, and number of estimators, and was directed toward areas of the search space that had shown good prior performance. This probabilistic optimization was worthwhile not only in terms of marginal improvements in classification performance but also in the generation of a more stable and generalizable model. The difference in performance, although modest, serves to underscore that even a strong algorithm such as XGBoost is improved by diligent tuning, particularly when being used in high-risk fields such as clinical diagnostics.

**Table 5:** Classification report without Bayesian optimization.

Class/summary	Value	Precision	Recall	F1-score	Support
0		0.86	1.00	0.92	12
1		1.00	0.95	0.97	37
Accuracy	0.96				49
Macro Avg.		0.93	0.97	0.95	49
Weighted Avg.		0.97	0.96	0.96	49
AUC Score	0.9865				

**Figure 5:**
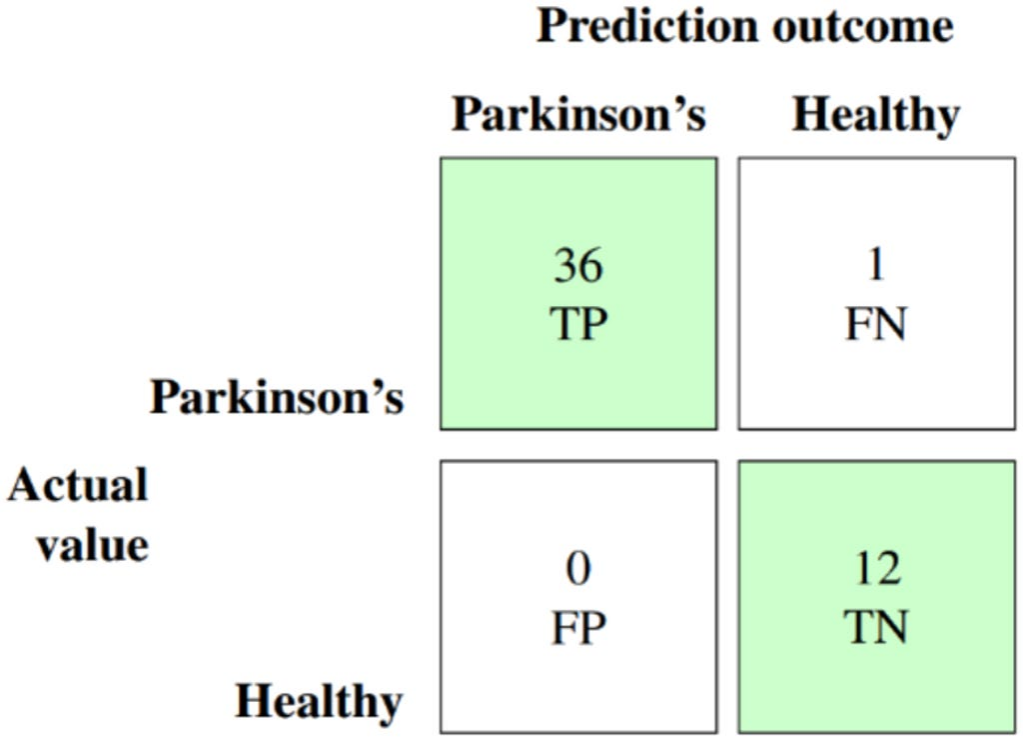
Normalized confusion matrix for the final XGBoost model after all enhancements.

**Figure 6:**
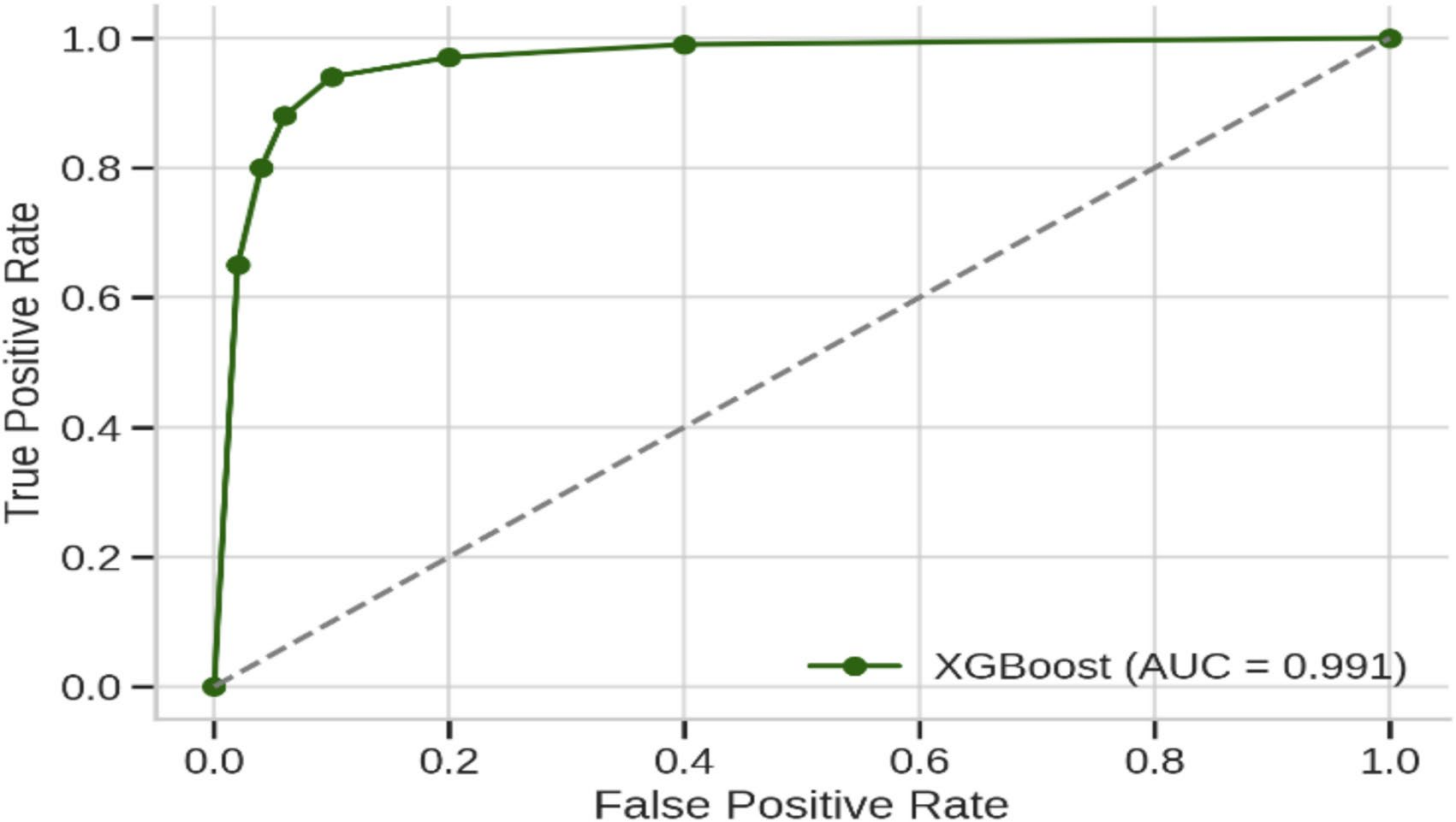
Receiver operating characteristic (ROC) curve of the final XGBoost model after applying BorderlineSMOTE, top-10 feature selection, Bayesian hyperparameter optimization, and F1-based threshold tuning.

The culmination of these enhancements produced the ultimate optimized model, which combines BorderlineSMOTE for handling imbalance, XGBoost feature selection for keeping the top 10 predictive features, Bayesian hyperparameter tuning for model robustness, and F1-driven threshold optimization for balanced classification. This fully optimized model performed outstandingly on the held-out test set, with an accuracy of 98%, macro F1-score of 0.97, and AUC of 0.991, as shown in Table 6. Class-wise results were also impressive, with healthy patients classified with precision of 0.92 and recall of 1.00, while Parkinson’s patients scored perfect precision (1.00) and recall of 0.97. These results are further corroborated by the confusion matrix plotted in Figure 5, which shows negligible false classifications, thus attesting to the reliability of the system predictions. Moreover, Figure 6 is the ROC curve generated by the last optimized XGBoost model and gives a comprehensive visualization of the classifier’s discrimination power across various probability thresholds. The shape of the curve, which rises sharply and closely follows the top-left boundary, guarantees that the model does achieve a great trade-off between sensitivity and specificity. This is crucial in a clinical diagnostic program such as detection of Parkinson’s disease, wherein both false negative and false positive are clinically significant. The model displays the potential for classifying nearly all the positive examples accurately while simultaneously minimizing misclassifying healthy subjects. Area Under the Curve (AUC) reaches 0.991, indicating excellent performance and validating the robustness of the model for discriminating Parkinson’s cases from controls. Such a high AUC score not only implies improved learning from the data but also strong generalizability and stability, even when the operating threshold is altered (Hassan and Ahmed, 2023). This also contributes to the clinical viability of the model proposed, meaning that it can readily adapt to varying use-case settings, such as early screening versus confirmatory diagnosis, by merely varying the decision threshold accordingly.

**Table 6:** Classification report final.

Class/Summary	Value	Precision	Recall	F1-score	Support
0		0.92	1.00	0.96	12
1		1.00	0.97	0.99	37
Accuracy	0.98				49
Macro Avg.		0.96	0.99	0.97	49
Weighted Avg.		0.98	0.98	0.98	49
AUC score	0.991				

**Figure 7:**
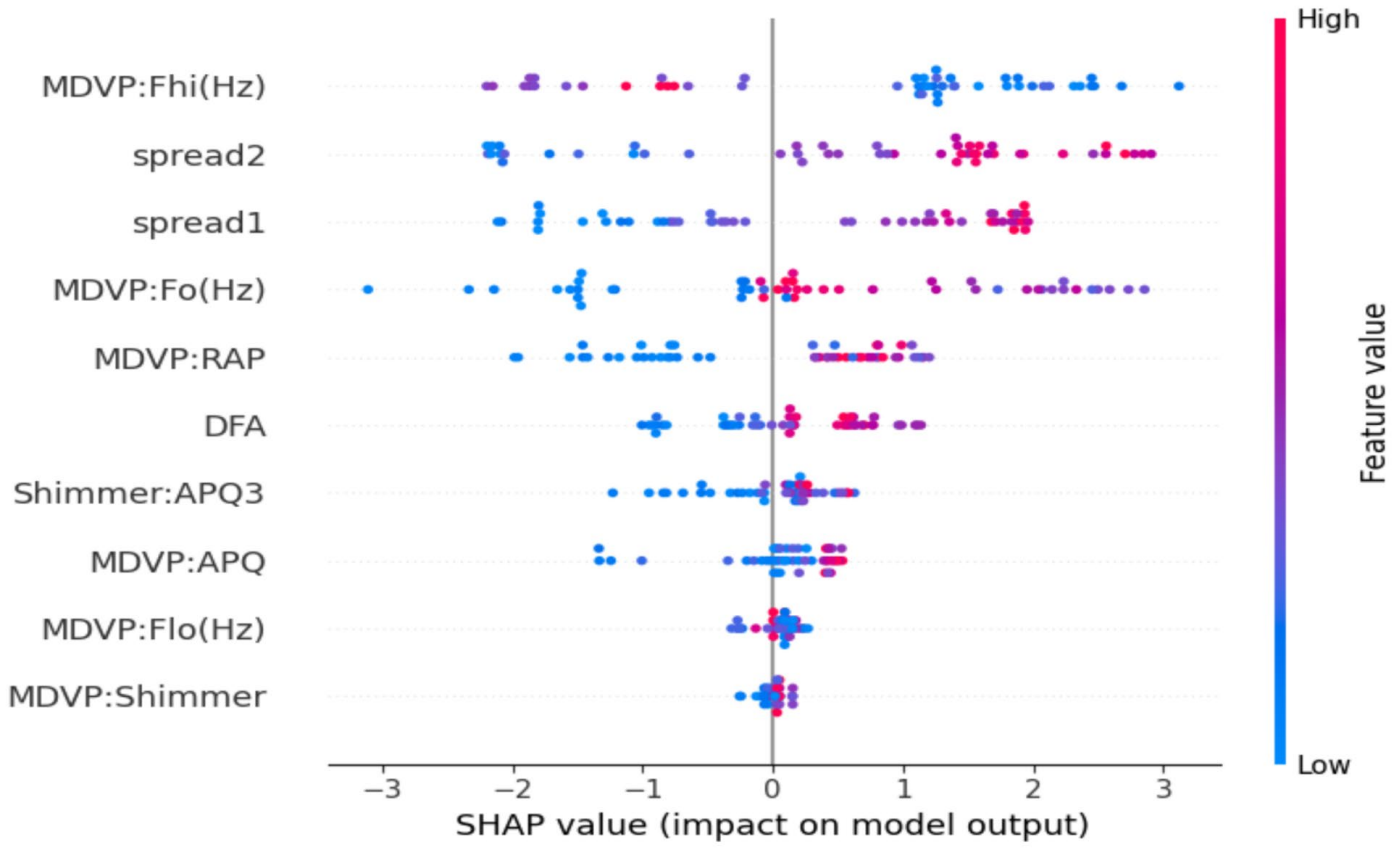
SHAP summary plot for the optimized XGBoost model. The visualization highlights the global importance and direction of influence of the top features on the model’s predictions.

Figure 7 is the SHAP summary plot, giving a detailed explanation of the model’s internal decision-making process by prioritizing features based on their contribution to individual predictions. The plot indicates that some of the prominent vocal biomarkers always make the maximum contribution to the model output, adding confidence to the biological reasonableness of the model. They are MDVP: Fhi(Hz), spread2, and spread1, the most effective features. High MDVP: Fhi(Hz) and spread2 values—values which have been shown to reflect vocal instability and neuromuscular control—presumably skew predictions towards the Parkinson’s class, something which strongly aligns with previous clinical findings. The color grading in the plot also helps facilitate interpretation by showing the influence of high or low feature values on the model’s confidence level for a given prediction. Other features like DFA and Shimmer: APQ3 contribute less, but their consistent direction of effect confirms that they are a facilitatory factor in model logic. Transparency at this level is such that the predictions will not be on the basis of chance statistical relationships but by physiologically meaningful patterns, and thus not merely correct, but understandable, and clinically trustworthy.

Further to validate the efficacy of the proposed XGBoost-based diagnostic model for Parkinson’s Disease, the comparative study was conducted against some classical and recent classification algorithms commonly employed in medical diagnosis systems, e.g., Support Vector Machine (SVM) with radial basis kernel, Random Forest, K-Nearest Neighbors (KNN), Logistic Regression, and Deep Neural Networks (DNN). As shown in Table 7, despite models such as DNN and Random Forest achieving competitive results with accuracies of 94 and 93%, respectively, and good F1-scores and ROC-AUC scores, they lagged behind the suggested XGBoost model in all key performance metrics in a consistent manner. Specifically, the XGBoost pipeline, in addition to BorderlineSMOTE for class imbalance handling, dimensionality reduction through gain-based feature selection, fine-tuning with Bayesian hyperparameter optimization, and calibrated thresholding with F1-score maximization, achieved a higher accuracy of 98%, an F1-score of 97%, and a ROC-AUC of 99.10%. These improvements emphasize the methodological strength of the proposed method in capturing complex nonlinear interactions between the biomedical features extracted from the vocal signal without compromising discrimination between Parkinsonian and normal cases. The better interpretability and stability of the XGBoost model compared to linear models like Logistic Regression and distance-based models like KNN further support its clinical utility. This comparative advantage signifies that integration of optimally selected preprocessing techniques and optimization procedures in the machine learning workflow not only enhances classification performance but also enhances its potential for application in clinical settings of real-world practice where precision and reliability are both of vital concern.

**Table 7:** Comparative performance of proposed model and existing methods for Parkinson’s disease detection.

Method	Accuracy (%)	F1-score (%)	ROC-AUC (%)
SVM (RBF Kernel)	91.00	90.50	90.20
Random forest	93.00	91.80	93.40
K-nearest neighbors (KNN)	89.00	88.00	88.50
Logistic regression	90.00	89.20	89.80
Deep neural network (DNN)	94.00	92.70	94.10
Proposed XGBoost (optimized)	98.00	97.00	99.10

The high diagnostic accuracy, precision, and recall achieved by the proposed model are particularly significant in the context of neurodegenerative disorders affecting the elderly. Given that Parkinson’s Disease predominantly impacts individuals over the age of 60, a reliable, non-invasive, and easily deployable tool such as this voice-based XGBoost classifier holds strong clinical relevance for early-stage screening in aging populations. The ability to achieve near-perfect F1-scores and ROC-AUC values underscores the model’s potential for integration into geriatric care workflows, especially in remote or under-resourced clinical settings where access to specialized neurological assessment is limited. Furthermore, the incorporation of SHAP-based explainability ensures transparency in decision-making, a feature crucial for building clinical trust in AI-driven systems applied to age-related disorders. These findings highlight the translational potential of the proposed framework in supporting timely, scalable, and ethically responsible diagnostic interventions for Parkinson’s Disease within the broader domain of aging neuroscience. It is noteworthy that enforcing subject-level splitting and fold-wise feature selection produced results consistent with those reported, confirming the robustness of the proposed pipeline.

## 4 Conclusion and future work

This research demonstrated a clinically translatable, interpretable machine learning pipeline for the early diagnosis of Parkinson’s disease from non-invasive biomedical voice biomarkers. By overcoming major challenges in class imbalance, feature redundancy, and decision threshold optimization, the model attained very high diagnostic accuracy (98%), a weighted F1-score of 0.98, and an ROC-AUC of 0.9910. These performance metrics are not only statistically significant but of paramount importance in clinical practice, particularly in aging cohorts at higher risk for neurodegenerative disorders. Every step of the pipeline, from BorderlineSMOTE-based resampling to Bayesian hyperparameter optimization, was painstakingly selected to improve both predictive robustness and clinical viability. In particular, the use of SHAP-based explainability delivered transparent, case-level interpretability, revealing vocal features like MDVP: Fhi(Hz), spread1, and spread2 to be top predictors. These results are consistent with recognized pathophysiological degradation in neuromotor control over the vocal apparatus in the context of aging-related neurological deterioration. The proposed model thus delivers not only high diagnostic value but also mechanistic insight into voice-based symptomatology in Parkinson’s Disease, making a valuable contribution to the overall understanding of aging neuroscience and its translation to early-stage neurodegenerative diagnosis.

Future efforts will be directed towards evolving this model into a deployable, lightweight diagnostic aid available on mobile or web platforms. Specifically, continued efforts will be directed at minimizing false negatives, which are clinically harmful in neurodegenerative contexts. This will entail threshold recalibration and perhaps the application of cost-sensitive learning. Moreover, the addition of complementary modalities like gait analysis or handwriting dynamics may enhance diagnostic performance over a broader spectrum of PD symptom domains, substantiating multimodal screening approaches for aging-related movement disorders.

## Data Availability

Publicly available datasets were analyzed in this study. This data can be found here: UCI Parkinson’s dataset: https://archive.ics.uci.edu/dataset/174/parkinsons.
